# A journey with Maffucci syndrome: From skull base chondrosarcoma to multiorgan management: A case report and literature review

**DOI:** 10.1016/j.radcr.2025.04.027

**Published:** 2025-05-02

**Authors:** Jana Dibas, Aseel Eid, Somaya Al Kiswani, Zaid Sawaftah, Nader Sarhan, Abdullah Nofal, Omar Sawafta, Jehad Khamaysa

**Affiliations:** a Department of Medicine, An Najah National University, Nablus, Palestine; b Radiology Department, King Hussein Cancer Center, Amman, Jordan; c Department of Radiology, Tubas Turkish Governmental Hospital, Tubas, Palestine

**Keywords:** Maffucci syndrome, Skull base chondrosarcoma, Enchondroma, Hemangioma

## Abstract

Maffucci syndrome is a very infrequently occurring genetic disorder. The 2 classic findings are enchondromas and hemangiomas with a high propensity to become malignant, leading to the formation of chondrosarcomas. In this study, we present the case of a 30-year-old male patient diagnosed with chondrosarcoma at the base of the skull related to Maffucci syndrome who presented with disturbances in visual perception and a palsy of the cranial nerve VI. His imaging studies confirmed the diagnosis; treatment included subtotal resection followed by radiation therapy. The following is the case that epitomizes the dreaded complications of Maffucci syndrome and the need for multidisciplinary, attentive follow-up to find early signs of malignant transformation.

## Introduction

Maffucci syndrome is a rare disorder characterized by multiple benign cartilage tumors, known as enchondromas, along with vascular anomalies such as hemangiomas. This condition is frequently associated with mutations in the IDH1 or IDH2 genes, leading to aberrant enzyme function. The presence of enchondromas weakens the bones, predisposing affected individuals to skeletal deformities and an increased risk of fractures. Additionally, Maffucci syndrome carries a substantial risk of malignant transformation, most notably into chondrosarcomas, which can develop in various locations, including the skull base. This case report highlights a patient with Maffucci syndrome who developed a chondrosarcoma at the skull base, emphasizing the potential for severe complications associated with this condition [1,2].

We present the case of a 30-year-old male diagnosed with Maffucci syndrome who developed a skull base chondrosarcoma. His medical history was notable for multiple enchondromas and hemangiomas, prior surgical interventions, and progressive visual disturbances. Imaging studies revealed a skull base mass alongside additional skeletal lesions. Management included surgical resection, adjuvant radiation therapy, and ongoing surveillance for disease progression, given the widespread nature of his enchondromas and hemangiomas.

## Case presentation

A 30-year-old male with a known history of Maffucci syndrome presented with right-sided blurred vision and right cranial nerve VI palsy. His past medical history was significant for multiple enchondromas and hemangiomas, as well as prior surgical interventions involving the right hand, left hip, and right shoulder. His surgical history included a right-hand core biopsy and multiple excisions of left-hand lesions. Histopathological examination of these specimens confirmed a low-grade cartilaginous tumor consistent with enchondroma in a right fifth finger mass and benign vascular neoplasms consistent with hemangiomas in multiple left-hand lesions.

The patient’s social history revealed that he was married, childless, and worked as an engineer. He was a former smoker and had no significant family history of malignancies. He reported no known drug or contrast allergies.

Laboratory results revealed minimally elevated uric acid levels at 8.2 mg/dL (normal range: 3.5-7.2 mg/dL), suggesting possible gout or renal involvement. Additionally, prothrombin time (PT) was slightly prolonged at 14.5 seconds (normal range: 11-13.5 seconds) and activated partial thromboplastin time (aPTT) was 39.8 seconds (normal range: 25-35 seconds), indicating mild coagulation abnormalities.

On physical examination, the patient appeared in good general condition. Neurological assessment confirmed right cranial nerve VI palsy, correlating with his complaints of blurred vision and diplopia and consistent with chondrosarcoma involvement of the skull base. A comprehensive cranial nerve examination is essential in such cases to evaluate for additional neurological deficits that may arise from tumor compression. Cutaneous findings included a laparotomy scar, a left-sided chest wall mass suspected to be an enchondroma, a right shoulder scar, and multiple scars and nodules on the hands and feet, consistent with hemangiomas.

**Fig. 1:**
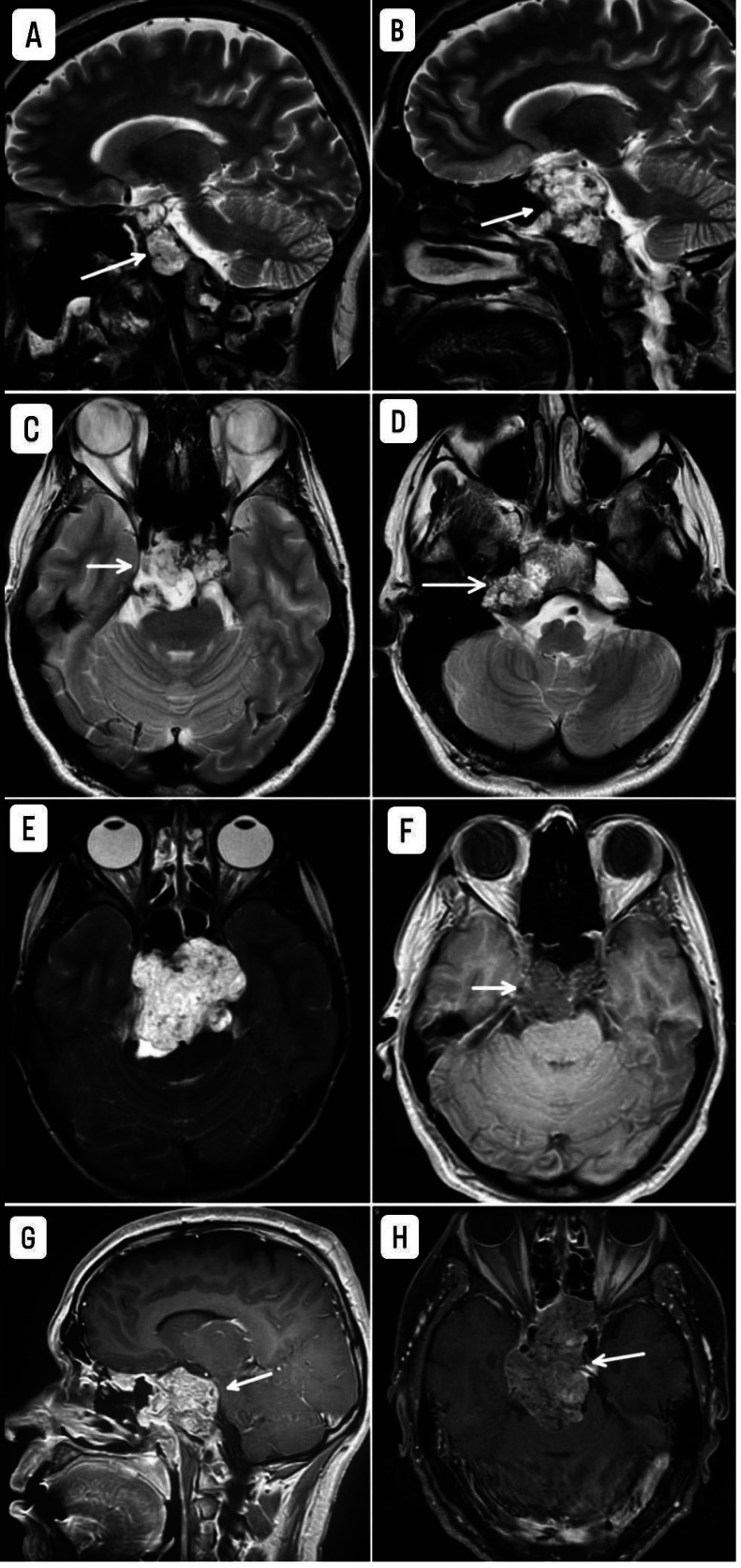
MRI findings of the skull base mass. (A, B) Sagittal T2-weighted images; (C, D, E) Axial T2-weighted images; (F) Axial T1-weighted image; (G) Sagittal T1-weighted image with contrast; (H) Sagittal T1-weighted image with contrast and fat saturation. The images depict an expansile mass lesion centered at the clivus and petrous apices, extending into the prepontine cistern, where it causes mild abutment of the right anterolateral pons and basilar artery. The lesion exhibits a heterogeneous T2 signal intensity and demonstrates heterogeneous contrast enhancement. There is bilateral cavernous sinus involvement, more pronounced on the left, where the lesion results in obliteration of the left Meckel’s cave. Superiorly, the mass extends to closely approximate the optic chiasm, raising concerns for potential visual pathway compromise.

**Fig. 2:**
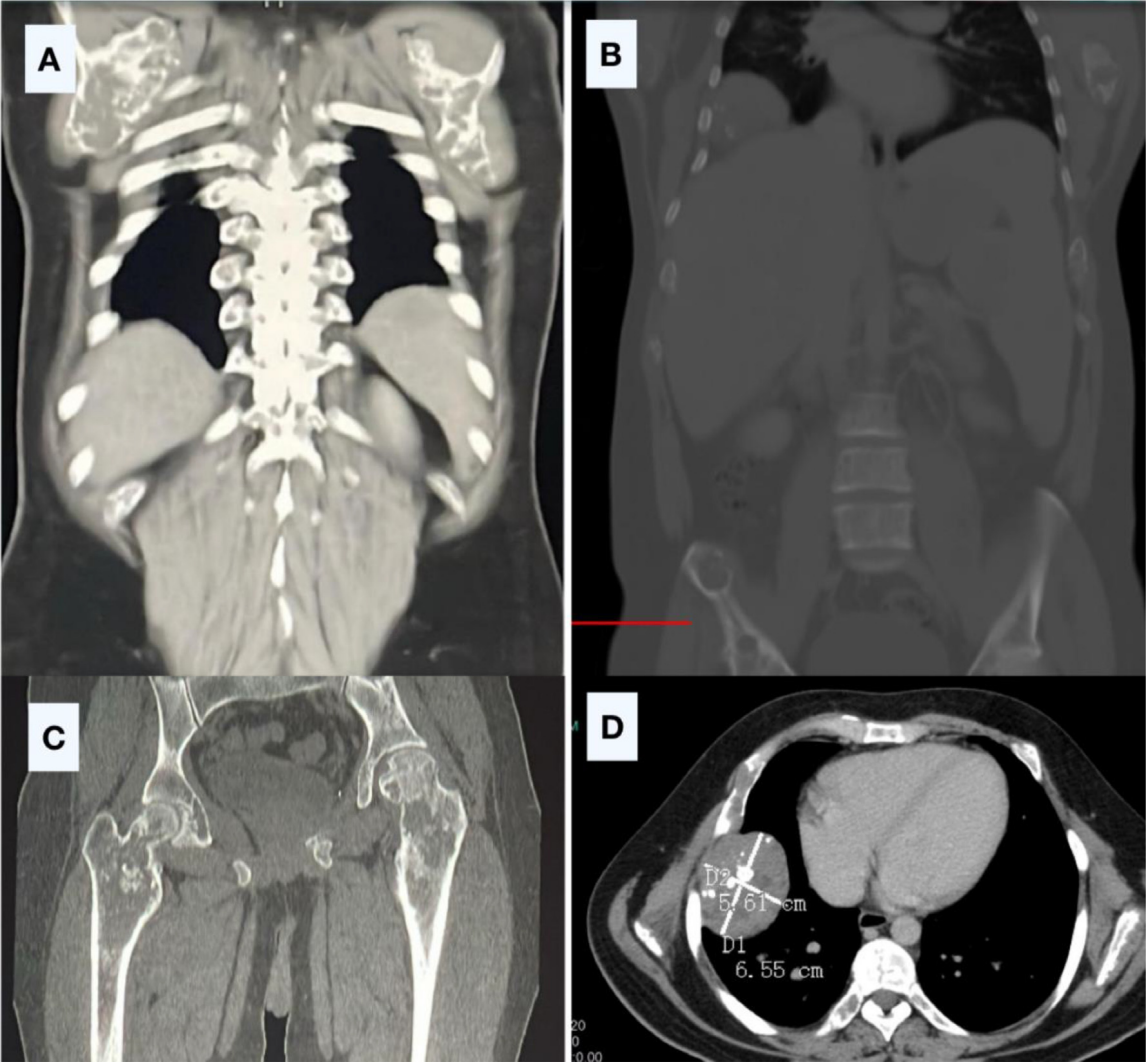
CT Chest, Abdomen, and Pelvis Findings: Multiple pulmonary nodules, concerning for metastases. Expansile lytic lesions with soft tissue components involving both scapulae (A, soft tissue window). Expansile lytic lesions in the pelvic bones, ribs, and scapulae (B, E, bone window). Multiple lytic lesions in both femur and the visualized pelvic bones (C). A large subpleural soft tissue lesion involving the right sixth rib, suspicious for malignant degeneration of an enchondroma (D).

**Fig. 3:**
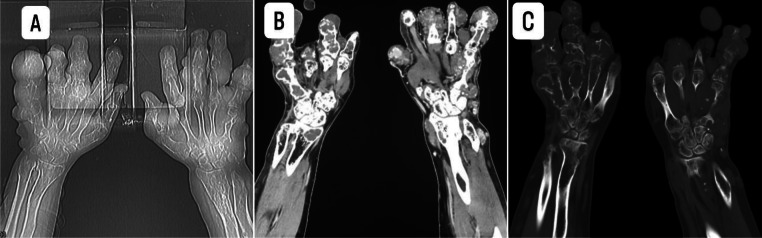
The scout X-ray (A) reveals extensive bilateral bony destruction involving the metacarpals, phalanges, and distal radii. There is irregular deformity and deviation of the distal radius, more pronounced on the right side, along with multiple expansile destructive bone lesions, consistent with enchondromas. The soft tissue window (B) demonstrates the presence of soft tissue components associated with the bony lesions. Additionally, multiple hemangiomas are noted within the surrounding soft tissues. The bone window (C) highlights extensive intramedullary expansile destructive lesions involving the metacarpals, phalanges, and distal radii/ulnae bilaterally. There is significant cortical thinning, with multifocal areas of cortical breakthrough, further supporting the diagnosis of multiple enchondromas with possible malignant transformation.

**Fig. 4:**
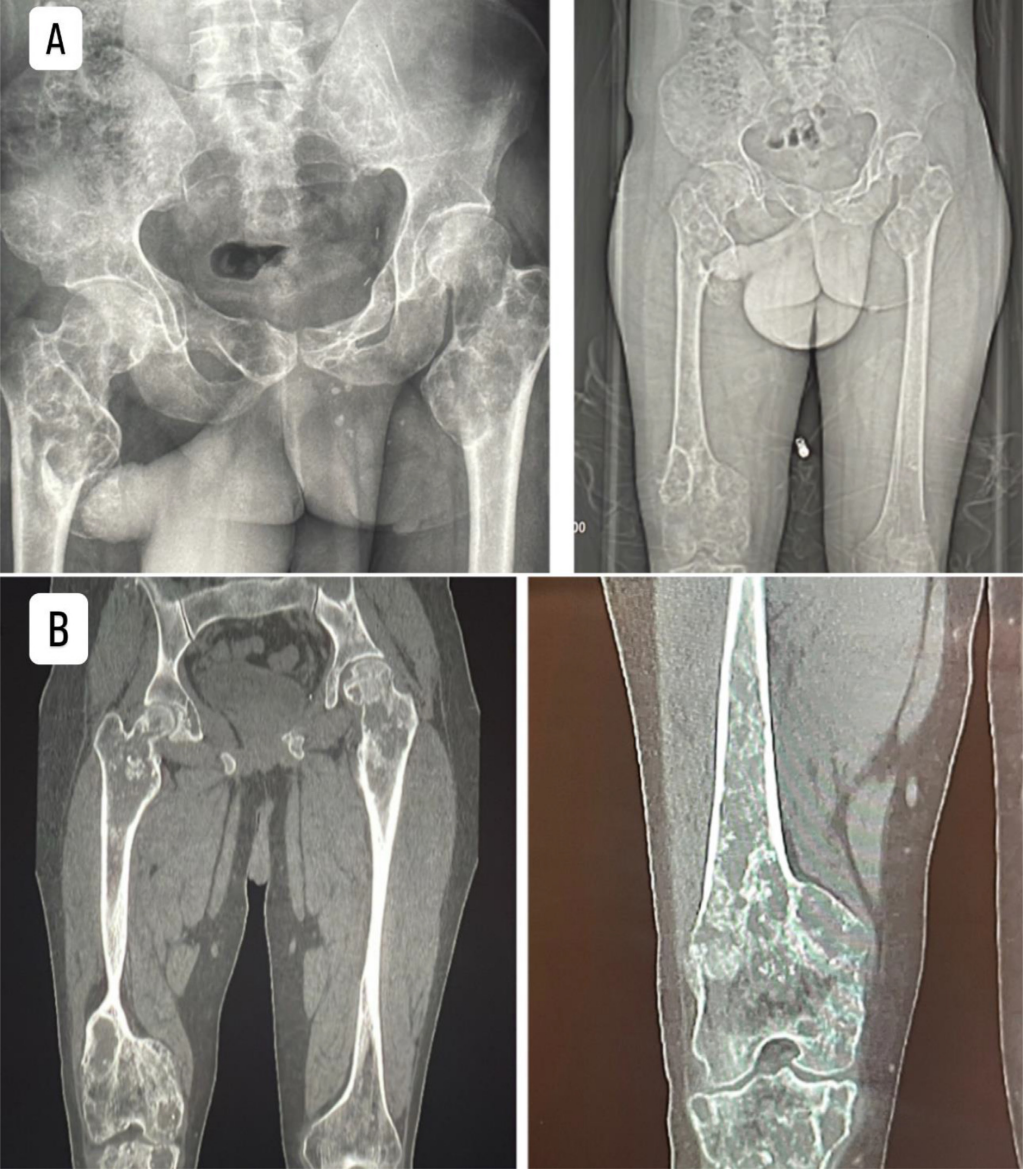
The scout X-ray and pelvic radiograph (A) demonstrate multiple lytic lesions involving the pelvis, proximal femora, iliac bones, and distal femora. These lesions appear expansile and destructive, with cortical thinning and areas of cortical breakthrough, consistent with multiple enchondromas. The bone reconstruction image (B) provides a detailed visualization, confirming the presence of extensive intramedullary lytic lesions in the proximal and distal femur, iliac bones, and pelvic structures. There is significant bone remodeling and cortical destruction.

Imaging studies revealed a skull base lesion consistent with chondrosarcoma (Fig. 1), a malignant rib lesion with suspected metastasis, multiple skeletal lesions characteristic of enchondromas, splenic abnormalities likely representing hemangiomas (Fig. 2), extensive bilateral destructive bone lesions in the hands and forearms (Fig. 3), and scout X-ray and pelvic radiograph (Fig. 4).

Further investigations included flexible endoscopy, which identified a right anterior two-thirds hemangioma in the posterior two-thirds of the oral tongue and a left base of tongue mass extending to the left vallecula. Additionally, histopathological analysis of various biopsies and excisions was performed. A testicular ultrasound revealed a large encysted hydrocele on the left side and a small hydrocele on the right.

The patient underwent subtotal/partial endoscopic resection of the skull base chondrosarcoma, followed by postoperative radiation therapy to the nasopharynx (70 Gy/35 fractions via volumetric modulated arc therapy [VMAT]). Additional interventions included multiple excisions of left-hand lesions and a right-hand core biopsy. The patient also reported using camel milk as part of his medical management, which he believed contributed to improved joint mobility and the regression of some masses. Ongoing management includes follow-up imaging with a repeat MRI of the neck to assess disease progression and response to therapy.

## Discussion

Maffucci syndrome is a rare, nonhereditary genetic disorder characterized by the presence of multiple benign cartilaginous tumors (enchondromas) and vascular anomalies, including hemangiomas and lymphangiomas [1].

The majority of Maffucci syndrome cases are attributed to somatic mutations in the IDH1 gene, with a smaller proportion linked to IDH2 mutations. These genes encode isocitrate dehydrogenase 1 and 2, enzymes responsible for catalyzing the conversion of isocitrate to 2-ketoglutarate, a process that generates NADPH, which is essential for various cellular functions. Dysfunction of these enzymes due to pathogenic mutations has been identified in cells of enchondromas and hemangiomas; however, the precise mechanisms linking these mutations to the clinical manifestations of Maffucci syndrome remain poorly understood [2].

Enchondromas in Maffucci syndrome typically arise asymmetrically in long bones, leading to skeletal fragility, bowing deformities, and an increased risk of fractures. Affected individuals may also present with limb length discrepancies, often detected during childhood. Notably, enchondromas in Maffucci syndrome carry a significant risk of malignant transformation into chondrosarcomas, with the likelihood increasing in proportion to the number of enchondromas. The estimated frequency of malignant transformation ranges from 15% to 40% [3].

Chondrosarcomas in patients with Maffucci syndrome can develop in various anatomical sites, including the trachea [4], nasal cavity [5], and skull base [6,7]. In this report, we present a case of a 30-year-old male with Maffucci syndrome who developed a chondrosarcoma at the skull base.

The patient had been diagnosed with Maffucci syndrome in early childhood and had undergone multiple surgical procedures for the resection of enchondromas and hemangiomas, including surgeries involving the right hand, left hip, and right shoulder. His initial symptoms of skull base chondrosarcoma included right-sided blurred vision and cranial nerve VI palsy. Imaging studies revealed a large petroclival bony mass consistent with chondrosarcoma. Management consisted of subtotal endoscopic resection followed by postoperative radiation therapy, which led to significant improvement in both symptoms and visual function.

Although chondrosarcomas are typically slow-growing, they can exhibit locally aggressive behavior. Thus, optimal management requires a combination of surgical resection and adjuvant radiotherapy to maximize tumor removal and minimize recurrence risk [8].

The management of Maffucci syndrome necessitates a multidisciplinary approach, with regular monitoring by orthopedic surgeons and dermatologists to assess skeletal and cutaneous changes, along with annual evaluations for malignant transformation. While no definitive cure exists, surgical intervention remains the cornerstone of treatment, alleviating symptoms and addressing complications as they arise [9].

The prognosis of patients with Maffucci syndrome complicated by chondrosarcoma is highly variable and depends on factors such as tumor burden, completeness of surgical resection, and metastatic spread. Low-grade chondrosarcomas are associated with favorable outcomes, whereas high-grade tumors exhibit increased aggressiveness, a higher propensity for metastasis, and a greater likelihood of recurrence [8]. In the absence of malignant transformation, individuals with Maffucci syndrome generally have a normal life expectancy [3].

## Conclusion

This case outlines the difficulty in managing Maffucci syndrome, since it carries a great risk for serious complications, including malignant transformation into chondrosarcomas. Combination treatment of surgical resection and radiation therapy effectively controlled the skull base tumor and the patient's symptoms. Regular follow-up and multidisciplinary follow-up are very important features in monitoring disease progression, tackling complications, and the early detection of malignancies. While no cure exists, early identification and comprehensive management can highly improve outcomes in patients affected with Maffucci syndrome.

## Patient consent

Written informed consent was obtained from the patient himself for his anonymized information to be published in this article.

## Ethics approval

Our institution does not require ethical approval for reporting individual cases or case series.
